# It’s not just a knee, but a whole life: A qualitative descriptive study on patients’ experiences of living with knee osteoarthritis and their expectations for knee arthroplasty

**DOI:** 10.3402/qhw.v11.30193

**Published:** 2016-03-31

**Authors:** Josefina Nyvang, Margareta Hedström, Sissel Andreassen Gleissman

**Affiliations:** 1 Department of Clinical Science, Intervention and Technology, Division of Orthopedics and Biotechnology, Karolinska Institutet, Stockholm, Sweden; 2 Department of Orthopedics, Karolinska University Hospital, Stockholm, Sweden; 3 Sophiahemmet University, Stockholm, Sweden; 4 Department of Clinical Sciences, Danderyd Hospital, Karolinska Institutet, Stockholm, Sweden

**Keywords:** Knee osteoarthritis, knee arthroplasty, qualitative research, experiences, expectations, pain

## Abstract

**Aim:**

Knee arthroplasties are an increasingly common treatment for osteoarthritis (OA) and the main indication is pain. Previous research states, however, that 15–20% of the operated patients are dissatisfied and 20–30% have persistent pain after surgery. This study is aimed at describing patients’ experiences of living with knee OA when scheduled for surgery and further their expectations for future life after surgery.

**Methods:**

We interviewed 12 patients with knee OA scheduled for arthroplasty, using semi-structured qualitative interviews. The interviews were recorded and transcribed *verbatim* and analyzed using qualitative thematic analysis.

**Findings:**

Three categories were formulated with an overriding theme: “It's not just a knee, but a whole life.” The three categories were “Change from their earlier lives,” “Coping with knee problems,” and “Ultimate decision to undergo surgery.” The main finding was that knee OA affects the whole body and self, ultimately affecting the patients’ lives on many levels. Further findings were that knee OA was considered to be the central focus in the participants’ lives, which limited their level of activity, their ability to function as desired, their quality of life, and their mental well-being. Although surgery was considered to be the only solution, the expectations regarding the outcome differed.

**Conclusions:**

The participants were forced to change how they previously had lived their lives resulting in a feeling of loss. Thus, the experienced loss and expectations for future life must be put into the context of the individual's own personality and be taken into account when treating individuals with knee OA. The experience of living with knee OA largely varies between individuals. This mandates that patients’ assessment should be considered on individual basis with regard to each patient.

Osteoarthritis (OA) is the most common joint disease among older adults, defined as cartilage destruction and rebuilding of bone close to the joint. Pain, loss of function, and disability are known consequences of OA (Pouli, Das Nair, Lincoln, & Walsh, 2014) and pain is the main indication for knee arthroplasty. However, there is a discrepancy between structural changes of the joint and patient-reported pain (Lundblad et al., 2011). In the United States, 4.2% of the population above 50 years of age live with an artificial knee joint and over half of people living with diagnosed OA will undergo a knee arthroplasty (Weinstein et al., 2013). Osteoarthritic pain is known to cause severe distress in the patients’ lives and the expectations regarding knee arthroplasties have been shown to be high or very high in the majority of the patients (Koenen et al., 2014; Pouli et al., 2014). However, 15–20% of the patients are dissatisfied with their replaced joint, experiencing a lack of function and limited walking ability, and as many as 20–30% have persistent pain, leading to reduced working ability and quality of life (Wylde, Dieppe, Hewlett, & Learmonth, 2007). This could be regarded as a problem since the main indication for surgery is pain. Many quantitative studies and registers focus on long-term survival of the prosthesis, physical complications such as infections, loosening of the prosthesis, and reoperation, but the patient's perspective has not been sufficiently studied. Patients may experience a lack of function and persistent pain without having a prosthetic failure. In a previous study, patients reported good outcomes after knee arthroplasty when asked directly, but when encouraged to speak more freely, they expressed concerns about persistent pain and movement difficulties (Woolhead, Donovan, & Dieppe, 2005). This highlights the discrepancy between answers obtained from questionnaires and those given in interviews, and, consequently, the significance of qualitative research.

It is important to learn more about patients’ experiences of living with OA, as well as their expectations for a knee arthroplasty, to better understand how OA affects their lives and how they cope with the disease. The present study can add to our knowledge of what improvements are required to be able to treat these patients in the best possible way, which is in line with person-centered care.

## Aim

The purpose of this study was to describe patients’ experiences of living with knee OA when scheduled for surgery and further their expectations for future life after surgery.

## Patients and methods

### Study design

The study used a qualitative descriptive design with semi-structured interviews (Patton, 2015). Qualitative interviews assert that people are experts about their own lives and the best ones to report how they experience a particular event or phenomenon. The purpose of these qualitative interviews was to enter into people's subjective perspectives and to gain rich information about living with knee OA, what it is like, and thus understand patients’ needs and improve their treatment.

### Pre-understanding

There is a need to have varied expertise to be able to apply and reflect upon multiple perspectives on a phenomenon. The study group has different pre-understanding and experiences from clinical work in orthopedics and nursing as well as research for people living with knee OA and other chronic illnesses.

### Patients

Inclusion criteria were > 18 years of age, diagnosed with knee OA, scheduled for knee arthroplasty at Karolinska University Hospital, Stockholm, Sweden, and able to be interviewed in Swedish. The exclusion criterion was a previous knee arthroplasty. Purposeful sampling (Patton, 2015) was used to obtain variation in age and sex. Twelve patients who consecutively met the inclusion criteria were asked to participate by an orthopedic surgeon and none declined.

To provide rich descriptions of the participants and further describe their knee function, they completed the Knee Injury and Osteoarthritis Outcome Score (KOOS) questionnaire (Roos, Roos, Ekdahl, & Lohmander, 1998), which is a reliable knee-specific self-assessment questionnaire for assessing baseline function and changes over time in individuals with knee OA (Steinhoff & Bugbee, 2014). They also completed the Health-Related Quality of Life (HRQoL) questionnaire, EQ-5D, regarding perceived problems in different dimensions of HRQoL (Brooks, Jendteg, Lindgren, Persson, & Bjork, 1991) and pain according to the Visual Analogue Scale (VAS) (Price, McGrath, Rafii, & Buckingham, 1983). These measurements are regularly used in clinical practice by orthopedic surgeons to facilitate the assessment of the patients regarding knee symptoms, general condition, and pain. The participants were slightly younger than the average population scheduled for knee arthroplasty but had corresponding knee-related problems (SKAR, 2013). See Table I for demographic features.

**Table I:** Patient demographics.

Age, median (range)	65.7 (47–77)
Women, no. (all)	7 (12)
Total knee arthroplasty, no.	10
Unicompartmental knee arthroplasty, no.	2
Work, no.	
Retired	5
Working	7
KOOS, median (range)	
Pain	42 (25–64)
Symptoms	59 (25–75)
ADL	53 (29–82)
Sports and recreation	3 (0–35)
Quality of life	8 (2–43)
EQ-5D VAS, median (range)	75 (30–93)
Pain VAS, median (range)	44 (4–76)

Knee Injury and Osteoarthritis Outcome Score, KOOS, consists of five dimensions graded from 0 to 100 in a worst-to-best scale.

EQ-5D, consists of a VAS scale assessing the patients’ HRQoL from 0 to 100 in a worst-to-best scale.

Pain Visual Analogue Scale, VAS, grades the intensity of the pain of the affected limb from 0 to 100 where 0 is no pain and 100 worst possible pain.

### Data collection and procedure

An interview guide was developed from literature covering OA and chronic pain and illness, and from the authors’ expertise and pre-understanding. The topics were: the patients' experiences regarding pain and function, OA's influence on physical activity and social life, fears about undergoing surgery, and expectations regarding the outcome of surgery. Semi-structured interviews with open-ended questions, rather than leading questions, were used to get vivid descriptions on the topics. However, the interviewer was free to ask relevant probing questions to get in-depth understanding of the patients’ experiences and expectations, and the participants were free to introduce new topics relevant to their stories. Each interview started with the request, “Please tell me about your experiences of living with knee osteoarthritis.” From the fall of 2013 to the spring of 2014, JN interviewed the participants within a month before the scheduled surgery at a location of their preference and convenience. The interviews were recorded and lasted between 25 and 65 min (mean=39 min).

### Data analysis

For interpretation, the interviews were analyzed using qualitative thematic analysis (Patton, 2015). According to Patton (2015), qualitative thematic analysis can be used when the purpose is not only to describe the content of the interviews, but also to interpret the findings at a higher level by formulating themes. Qualitative thematic analysis consists of a descriptive part and an interpretive part (Patton, 2015). Each interview was transcribed *verbatim* by a research assistant and read through by the interviewer while listening to the recording to ensure closeness to the recording. In the first part of the analysis, the interviews were read several times by all authors, with the aim in mind, and notes were made in the margin to comprehend the overall meaning and to search for patterns. Key phrases (sentences or words) were identified separately by the authors and then compared and discussed back and forth to reach agreement and ensure the validity and trustworthiness of the analysis (Patton, 2015). The key phrases were coded, using highlighting pens, and thereafter structured into preliminary categories addressing similar contents. The coding process facilitated the discovery of recurring data in the interviews and ultimately the creation of categories. In part two, the categories were discussed and compared with an interpretive mind-set and the underlying meanings of each category were extracted. Based on the three categories, one overriding interpretative theme was formulated. See Table II for examples of the analysis procedure.

**Table II:** Examples of the analysis procedure.

*Key phrases*	Code	Subcategory	Category	Overriding theme
*You always have to plan what you do now, I never had to do that before*	Reduced spontaneity	Struggling through everyday life	Change from their earlier lives	It's not just a knee, but a whole life
*The pain takes away the joy of doing things*	Pain affects the mood	Emotional distress		
*I have to take the elevator instead of stairs*	Physically reducing pain	Physical coping strategies	Coping with knee problems	
*It doesn't get better just because I go around moaning*	Positive thinking	Cognitive coping strategies		
*I don't take many pills, I only do it when nothing else works anymore*	Reluctance to taking painkillers	Alleviating pain		
*For now I feel that it's getting worse and worse and I want to put an end to that*	Surgery as the only way out		Ultimate decision to undergo surgery	

### Ethical considerations

All participants were informed of the voluntary participation and guaranteed confidentiality and gave their written informed consent in accordance with the Declaration of Helsinki (WMA 2013). The study was approved by the Regional Ethical Review Board at Karolinska Institutet Stockholm, Sweden, Dnr 2012/786-31/2.

## Findings

One overriding theme: It's not just a knee, but a whole life, was created with three underlying categories: Change from their earlier lives, coping with knee problems, and the ultimate decision to undergo surgery. Representative quotes from the interviews illustrate the findings.

### It's not just a knee, but a whole life

Living with knee OA meant becoming aware of an aching body part, which affected the participants’ mood and life. They had to cut down on physical activity, which created a feeling of loss since they had to give up something they considered to be part of their normal life. Social relationships were affected when they had to decline certain activities. Altogether, they experienced a restricted life with less spontaneity. They often dreamed about regaining their previous level of physical activity, but the knee was an obstacle to achieve these dreams. However, they developed mental and physical strategies to handle their limitations. When strategies became insufficient and the pain unbearable, surgery was seen as the only way out. Being scheduled for surgery meant building up hope and expectations about their future life without pain and restrictions. Some hoped to be completely restored, while others simply expected the knee not to get worse. OA not only affected their knee, but their whole life.

### Change from their earlier lives

#### Struggling through everyday life

Many of the participants had previously been physically active, but, as the symptoms progressed, they were forced to cut back on these activities. Not being able to walk as they once used to was often experienced as being worst of all. Restricted leisure activities created a feeling of loss. Dreams of wandering, traveling, dancing, or running were common, but pain was the major deterrent to these activities. Pain made them tired and led to difficulties in concentrating, which made the joy of performing leisure activities vanish.

Pain and swelling could last for days after physical activities, which limited how they moved even more. This, in turn, limited their ability to be spontaneous.

You always have to plan what you do now, I never had to do that before. If I wanted to go biking, wanted to go skiing, or wanted to do something else, I could do it whenever I wanted to, but I can't do that now.

Many of the participants were working full-time, while others worked shorter days. At work, they had problems bending down or getting on their knees, which made them work at a slower pace.

I run the stores, and then you're up and about all day. When I'm at the store I've got energy, but when I get away from there … then I come home and don't want to move much. When you leave your workplace, you notice how you limp and then your leg is tired.

Other implications on daily living were difficulties in performing housework or managing their daily hygiene due to the heavy load on or uncomfortable movements of the knee that some tasks involved.

When I'm done in the laundry room my knee hurts really bad, as I reckon, due to the load of the laundry. That heavy load gives you more pain.

Pain at rest was common and could be more excruciating. It was alleviated by movement. Many had trouble falling asleep due to difficulties in finding a comfortable position without pain and woke up with severe pain, making it hard for them to go back to sleep. Thus, the quality of their sleep was affected, making them tired during the daytime.

But the pain often comes at night, so you wake up several times. Every time you move, you feel it, and it has also happened that the pain has been so intense that I have woken up and almost screamed. I had to bite the pillow because it hurt so much.

#### Emotional distress

Living with a constant awareness of an aching knee was mentally challenging and sometimes led to feelings of disappointment with themselves when they were not able to do what they wanted to. The pain made them irritable or even angry, which could distance them from their loved ones.

The pain takes away the joy of doing things. Because you don't get done what you imagined because it hurts too much, and then, (it) takes over and I get angry, and that doesn't help.

This sometimes led to a sense of feeling down and thoughts about life not being worth living.

So I'm a bit more depressed now than before, I think. Thanks to this … Well, I think, “Why should I go here when I just as well can die, and so on”; that's how I can think, “it's not a long time left,” and like that—those black thoughts I can get.

The participants felt inhibited and considered their knee as an obstacle toward the way of living as desired.

Fear was prevalent, especially a fear of falling due to immediate pain when walking on uneven ground or down slopes. Many of the participants had fallen several times, which made them more careful about how they used their knee.

Living with pain was experienced as mentally tiring and an obstacle to being socially active. The tiredness made them take daytime naps or forced them to be active constantly during the daytime to avoid falling asleep.

It's agonizing if you're attending meetings and the like. It's hopeless. Then the tiredness comes, and you fall asleep almost directly. It's a bit annoying. It has affected the work so to say.

Limping made the participants lag behind when walking together with others or have to stop abruptly, leading to difficulties in managing social events or meeting people. Some participants expressed concerns about how they were looked upon when walking.

But still, even in a sober state you are almost a little shaky when you walk, as if you weren't completely sober.

Some of the younger participants experienced a lack of understanding, especially by older people who, they believed, did not understand their situation. They considered their age to be a factor that contributed to the decline in their quality of life and gave rise to a feeling of being older than their chronological age.

And in my leisure time, if I'm going out shopping, sometimes I don't manage to keep up with the family; then I have to sit on some bench and tell the rest of the family that they can go into that shop and I'll wait here. So it affects the quality of life, and especially at this age. But it shouldn't be like that. I think, “you're going to have to sit like a pensioner on the bench. No!”

### Coping with knee problems

#### Physical coping strategies

The participants developed strategies to avoid pain by moving in new ways, by planning their journeys, or walking beforehand to avoid situations in which pain would be intolerable. They chose places with escalators or elevators, where there are benches to rest, or took cars instead of trains or buses.

I can't take long walks when I'm out walking, for example, because then I know that I will get pain, and especially if I walk in town where there's asphalt. That also affects (the knee), and it depends on what type of shoes I have on. Strangely enough, it's easier to walk in the woods, even though it's uneven, but it's soft. Otherwise, I can't dance and sometimes I have to take the elevator instead of stairs.

Walking on hard ground was painful and led to the use of soft shoes or walking beside the road in the grass. Walking down stairs or slopes was usually most painful, but they learned to put the least possible pressure on the knee, such as walking with serpentine movements or backwards and sideways. Pain often escalated the further they walked and was relieved when they sat down. While some still took walks, others had chosen to opt out longer walks or walk as little as possible. The use of crutches or knee-supporting band aids was sometimes required to be able to walk. To avoid exaggerated pain, they put more pressure on the healthy side. This, however, led to pain from joints on the other side, causing worries about affecting or damaging other joints.

#### Cognitive coping strategies

The participants handled their feelings and disabilities by creating a new mind-set to avoid thinking or caring about the pain. There was also a desire not to bother other people by talking about the pain.

I know that it hurts. There's nothing to do about it. It doesn't get better just because I go around moaning.

Some participants accepted the pain and had learned to live with it. Living with osteoarthritis could open people's eyes to those with similar problems, and even bring the realization that others may be even worse off.

But you notice, like, in the community, when you yourself have started to limp you look at people in a different way. And you actually see many that walk around limping.

The participants expressed stubbornness about continuing to live as they wanted to despite the incapacitating pain. Alternative therapy or working in a positive environment were external factors that facilitated living with OA.

#### Alleviating pain

Some participants could not manage without regular analgesics, while others used them when doing overstraining activities or as a last resort. There was a general fear that taking analgesics for a long time would be dangerous, and thus a striving to take the lowest doses possible. Even those who did not experience side effects were not keen about taking analgesics. However, feelings of being intoxicated, tired, unfocused, or noxious were also expressed.

No, I don't think it's any fun if I'm going out to play golf and I have to take painkillers and you don't have a proper focus either. No, that's also why I want to get rid of the painkillers. I don't take many pills, I only do it when nothing else works anymore.

Physiotherapy was rarely suggested by health care personnel and, therefore, few had undergone preoperative training. Some of those who had tried physiotherapy cited a worsening, while others reported short-term positive effects. Exercise on their own initiative resulted in improvements, for some, but worse pain for others. Training was believed to facilitate recovery after surgery.

### Ultimate decision to undergo surgery

Regarding when they decided on surgery, the participants described not being able to stand the pain any longer or being fed up with the situation.

Because now it's getting insufferable and it wasn't enough to take one Diclofenac pill if you were to have a good day. And then, that was when I called again (to the orthopedic clinic). It's the pain and that you can't walk. And that's limiting.

Other reasons were fears about a higher risk of a poor postoperative outcome if they postponed surgery, or progress of the symptoms continued. Many described having seen x-rays of their knee, which made them understand that OA could never self-mend and believe that surgery was the only way out of the misery.

My mother has told me, “Well, but… you have to do surgery,” and I've said like all the time, “No, it's not, I'm not there yet.” But it was likely that I didn't have any cartilage (left). That was probably what did it; then I thought, “No, now is the time.”

Family, acquaintances, and friends were sources of information about the surgical procedure, postsurgical management, and realistic expectations for the outcome of surgery. Both positive and negative experiences were influential, but in favor of the positive ones.

The decision to undergo surgery raised questions and fears about the procedure. Remaining awake during surgery was often dreaded. The magnitude of the operation itself made the participants realize that it could go wrong. Postoperative pain was anticipated, but it was believed to subside with time and physiotherapy. The participants expressed confidence in the surgeon's experiences and skills to achieve a positive outcome, but postoperative training was believed to be equally important. An information brochure, together with a model of an artificial knee, made them feel well informed and safe, and thus less nervous.

Expectations concerning the outcome of surgery were often related to dreams of their lives before OA with restored function. Some participants expected to be fully restored, while others expected the progress of the disease to stop.

For now I feel that it's getting worse and worse and I want to put an end to that. So that it won't keep getting worse. That's probably my hopes and it would also be my expectations.

Pain relief and being able to walk unimpeded were frequently described expectations. Furthermore, they hoped to take longer walks without worrying about lying awake due to pain. They dreamed about not having to plan ahead or constantly having the knee on their minds.

[I expect] to be able to walk. That it [the leg] will be like it used to be. That's what xx [the surgeon] told me, that I would be relieved, that I would be able to move the leg.

Expectations for performing leisure activities varied. To take long walks in the woods, go swimming, play golf, or jog were expectations they thought of as being reasonable, while running or jumping were activities they did not expect to manage. Replacing the knee was expected to decrease pain and symptoms, not only from the knee, but from other joints as well.

## Discussion

The main finding was that knee OA affects the whole body and self, ultimately affecting the patients’ lives on many levels. Further findings were that knee OA was considered to be the central focus in the participants’ lives, which limited their level of activity, their ability to function as desired, their quality of life, and their mental well-being. Although surgery was considered to be the only solution, the expectations regarding the outcome differed. The overriding theme, “It's not just a knee, but a whole life,” represents all levels affected.

As described in the literature (Hall et al., 2008; Pouli et al., 2014), pain was reported in our study to be a central focus when living with OA and to affect whole lives. The participants were constantly aware of their knee. We interpret this as a transition from a “lived body,” a unity between body and self, in which the participants did not think about the body, to an “object body,” when the knee, due to OA, revealed itself and caused a disruption between the unity of body and self (Gadow, 1980). Further, this can be interpreted as body awareness (BA), which was defined by Lööf, Johansson, Henriksson, Lindblad, and Bullington (2014) as “the tendency to focus attention on bodily internal sensations and stimuli.” Negative BA was, in our study, when the participants were not able to perform physical activities as before and positive BA when learning to live with the disease (Lööf et al., 2014). Our study highlights the importance of physicians treating the patient as a whole person, rather than only treating the knee, in order to understand the complexity and effects of OA. In the conceptualization of embodiment (Gadow, 1980), people learn to live with the new awareness, which is also the case in our study for the participants who developed cognitive strategies to cope with their pain. However, the awareness of the knee remained constant and progressive over time, which is consistent with earlier research findings (MacKay, Jaglal, Sale, Badley, & Davies, 2014).

The participants had varied perceptions about what they considered to be their normal level of physical activity. However, during the disease progression, they were all forced to change the way they had previously lived their lives. As described previously (MacKay et al., 2014), the participants reported not being able to perform leisure activities as before, which could be interpreted as a feeling of loss and, ultimately, an altered sense of self. Thus, the experienced loss must be put into the context of the individual's own personality (MacKay et al., 2014) and be taken into account when treating individuals with knee OA.

The disruption in physical activity had implications for social relationships when the participants had to abandon certain leisure activities related to such social events, which is in line with other studies (Demierre, Castelao, & Piot-Ziegler, 2011; Hall et al., 2008; MacKay et al., 2014). The lack of spontaneity, resulting from always having to plan ahead, has been described in several studies regarding OA and chronic illness (Egberg, Andreassen, & Mattiasson, 2012; MacKay et al., 2014). Egberg et al. (2012) examined how patients experienced living with peripheral artery disease, which can be compared to our study due to similarities in pain and mobilization. They concluded that performing specific activities and belonging to social networks contributed to individuals’ self-image. In our study, the participants dreamed about regaining their previous physical activity and going back to their normal lives. Not being able to live as before can be interpreted as a “lost self.”

The participants described both physical and mental tiredness due to pain and difficulties in sleeping, which may be interpreted as fatigue. This has been described in an earlier study as mental fatigue, which could be described as feeling drained, and physical fatigue, which is related more to aches and pain, ultimately affecting relationships since pain was suggested to be a powerful precursor to fatigue (Power, Badley, French, Wall, & Hawker, 2008). Our findings suggest that this type of tiredness, that is, fatigue, affects the participants’ lives on many levels and should be considered and assessed by physicians.

The participants struggled toward normalcy and developed both physical and mental strategies to handle living with OA, which has been described earlier in patients with severe chronic illness (Ohman, Soderberg, & Lundman, 2003). Although knee OA may not be regarded as a severe chronic illness, our findings show that the participants’ lives were greatly affected and generated thoughts not dissimilar to those of patients with severe illness (Ohman et al., 2003).

The participants expressed ambivalence about using analgesics. Fears of side effects and dependency have been discussed frequently in previous research (Alami et al., 2011; Pouli et al., 2014; Sale, Gignac, & Hawker, 2006). However, the participants in our study did not report a fear of dependency, but, instead, fear of long-term side effects. The reluctance to take analgesics should be investigated by physicians, and patients should be assessed individually regarding the risk of side effects and the predicted efficacy to be able to provide the best possible care and quality of life. Since pain was reported to be the core of the decline in physical and emotional well-being, better patient knowledge of pharmacological treatment options might benefit them. Physicians must regularly assess pain and medication compliance to prevent pain from causing a progressive decline in the patients’ quality of life.

As previously discussed, experiencing pain from a body part causes a disruption between the body and self, which affects the whole individual (Gadow, 1980). Pain itself should be assessed as a condition greatly affecting the individual and thus should be taken seriously. Furthermore, the radiographic grade of OA has been shown to correlate poorly with patients’ experienced pain (Lundblad et al., 2011), which could be regarded as a further incentive to address pain separately.

The decision to undergo surgery can be regarded as a breaking point, as described earlier (Hall et al., 2008). The combined consequences of limitations in functional mobility, leisure, and social activity, together with pain and inadequate pain relief, led to an understanding of the need for surgery, a breaking point (Hall et al., 2008). To be able to objectively verify the severity of the disease may positively affect the patients’ approach to surgery and eventually function as an empowerment allowing them to be part of the decision, rather than having it left for the surgeon only. In a previous study, the participants felt empowered if they were able to make decisions during the consultation and this helped them to manage their condition (Small, Bower, Chew-Graham, Whalley, & Protheroe, 2013). In contrast, Ong, Jinks, and Morden (2011) found that the participants felt empowered not only when managing illness but rather when learning to live with the disease by getting on with their daily life. However, the participants in their study, compared to ours, differed since they were not scheduled for knee arthroplasty and thus not had reached breaking point (Ong et al., 2011).

Expectations concerning the outcome of surgery were strongly related to what the patients considered they had lost during the progress of the disease, such as physical activity and unawareness of the knee. This may be related and interpreted as reuniting body and self and rebuilding a “lived body” rather than an “object body,” as described by Gadow (1980) and further interpreted by MacKay et al. (2014).

### Method discussion

A qualitative descriptive study design is suitable when the aim is to explore a phenomenon experienced by patients. We included 12 participants in our study, which was reasonable given the timeframe of the inclusion. One may discuss that 12 participants is a small number but we believe that the descriptions given by the participants were rich and exhaustive and that further inclusion would not add to the data. One limitation is that our findings cannot be generalized to describe a population. However, our aim was not to provide generalizable results but rather to describe the phenomenon of living with knee OA.

### Clinical implications

The experiences of living with knee OA varied among the participants, and we suggest that physicians, in order to be able to meet and treat patients in the best possible way, should assess patients as individuals. The patients may be able to take part in decision-making regarding the management of symptoms as part of patient empowerment and also be presented with different treatment methods that are most suitable for the patient's needs. Expectations regarding surgery may be surveyed and thoroughly discussed between the patient and the physician to mitigate unrealistic expectations.

## Conclusion

Knee OA affects the whole body and self, ultimately affecting the patients’ lives on many levels. The participants were forced to change how they previously had lived their lives resulting in a feeling of loss. Thus, the experienced loss and expectations for future life must be put into the context of the individual's own personality and be taken into account when treating individuals with knee OA. The experience of living with knee OA largely varies between individuals. This mandates that patients’ assessment should be considered on individual basis with regard to each patient.
